# Prevalence of Complications after Limberg Rhomboid Flap in Patients with Cutaneous Defects at Tertiary Care Hospital

**DOI:** 10.31729/jnma.4301

**Published:** 2019-04-30

**Authors:** Binod Bade Shrestha, Mikesh Karmacharya, Laxmi Bogati, Pradeep Ghimire

**Affiliations:** 1 Department of Surgery, Manipal College of Medical Sciences, Pokhara, Nepal

**Keywords:** *complications*, *cutaneous defects*, *limberg rhomboid flap*

## Abstract

**Introduction:**

Limberg rhomboid flap is an extremely useful and versatile technique to cover the cutaneous defects in various anatomical locations of different etiology and varied sizes. The main aim of the study is to find the prevalence of complications after limberg rhomboid flap in patients with cutaneous defects at a tertiary care hospital.

**Methods:**

This descriptive cross-sectional study was conducted at a tertiary care hospital from October 2015 to November 2018 after obtaining approval from the institutional review committee. Study population is patient admitted to ward and outpatient department of surgery. Convenience sampling was done. Data was entered and analyzed in statistical package for social sciences and point estimate at 95% confidence interval was calculated along with frequency and proportion for binary data.

**Results:**

Out of total patients, the complications were seen in total 8 (15.7%) patients. Prevalence of complications is 8 (15.7%) at 95% confidence interval (7.85–23.56). Among which, complications were seen in 5 (9.8%) bed sore, 2 (3.92%) in pilonidal sinus, 1 (1.96%) in traumatic ulcer and none in neoplastic lesion and types of complications seen were wound gaping in 3 (5.88%) cases, surgical site infection in 2 (3.92%) cases, recurrent pilonidal sinus in 1 (1.96%) case, flap necrosis in 1 (1.96%) case and epidermolysis in 1 (1.96%) case.

**Conclusions:**

The Limberg rhomboid flap can be used safely in patients with cutaneous defect with minimal complications and good surgical outcome however prevalence of complications after limberg rhomboid flap in patients with cutaneous defects at tertiary care center is high compared to the previous studies done.

## INTRODUCTION

The integumentary system which covers the entire surface of the human body may get affected by many conditions. If a cutaneous lesion is small it can be excised and closed primarily but it is not possible for larger lesions, for which skin grafts or local flaps are to be used.

The rhomboid flap, initially described by Alexander Limberg, can be used in almost every area of the human body for reconstruction of the cutaneous defects. The limberg rhomboid flap is done as it is one of the simple way of reconstruction with less failure rate and better outcome.

The main aim of the study is to find the prevalence of complications after limberg rhomboid flap in patients with cutaneous defects at tertiary care hospital.

## METHODS

This descriptive cross-sectional study was conducted in the Department of Surgery of Manipal Teaching Hospital, Pokhara, Nepal from October 2015 to November 2018 after obtaining approval from the institutional review board. Study population is patient admitted to surgery ward and patients of outpatient department of surgery. The patients aged 18 and above, with cutaneous defects of varied etiologies in different parts of the body were enrolled in the study. The patients below 18 years, immuno-compromised patients, those taking steroids and Type II diabetes mellitus were excluded from the study. All patients were assessed by age, etiology, site, size of defects and postoperative complications. Data was collected continuously during the study period. Convenience sampling was done and sample size was calculated using the formula,


$$\begin{array}{lll} \text{n} & = & {\text{Z}}^{2}\times (\text{p}\times {\text{q)/d}}^{2}\\ & & =1.96^{2}\times (0.09\times 0.91)/0.08^{2}\\ & & =49.14\\ & & =50 \end{array}$$

where,

n = sample size
p = prevalence from educated guess. (i.e. 9%)
q = 1-p
d = margin of error.
z = 1.96 at 95% CI.

After taking non-respondent rate 5%, the total sample size is calculated to be 53.

Surgeries were performed under local, spinal and general anesthesia with respect to size and site of disease. In cases of neoplastic lesions and pilonidal sinus excision was done first in order to create a defect. Whereas, in cases of traumatic and bedsore ulcer following the debridement a defect was conceptualized as rhomboid shape then the flaps were prepared with consideration of size and site of defect keeping in mind regarding the elasticity of adjacent tissue. While creatinga defect and flap, all angles were made of 60/120 degree meaning every side of defect and flaps were equal in length. ^1–3^ All excised defects were conceptualized as rhomboid shape with two equilateral triangles placed base to base. A line of same length as the base of triangle was drawn horizontally from the shorter diagonal of its own length. The second side of flap was marked with the line of same length as the first adjacent of the diamond creating 60 degree of the flap apex.

Cautery was used to excise the defect, undermining the subcutaneous tissue in order to stretch downwards without tension and maintaining hemostasis. Buried polyglactin of appropriate size were used to secure the flap in position. Placement of negative suction drain was done wherever necessary. Skin was sutured with 3/0, 4/0 prolene with vertical mattress and dressing was done.

Selection and information bias has been minimized as possible. Data entry was done and was analyzed in SPSS, point estimate at 95% CI was calculated along with frequency and proportion for binary data.

## RESULTS

Out of total patients, the complications were seen in total 8 (15.7%) patients at 95% CI (7.85–23.56). Among which, complications were seen in 5 (9.8%) bed sore, 2 (3.92%) in pilonidal sinus, 1 (1.96%) in traumatic ulcer and none in neoplastic lesion. The complications were minor except for one case of flap necrosis (Table 1).

**Table 1:** Rate of complications according to the type of lesion.

Cases	Complications n (%)
**Neoplastic**	**0 (0)**
**Traumatic**	**1 (1.96)**
**Pilonidal Sinus**	**2 (3.92)**
**Bed Sore**	**5 (9.8)**

Among the complications seen in patients, wound gaping in 3 (5.88%) cases, surgical site infection in 2 (3.92%) cases, recurrent pilonidal sinus in 1 (1.96%) case, flap necrosis in 1 (1.96%) case and epidermolysis in 1 (1.96%) case (Table 2). There were no incidents of hematoma, seroma and local tissue distortion.

**Table 2:** Type of complications in the patients.

Type of complications	Cases n (%)
Wound gaping	3 (5.88)
Surgical site infection	2 (3.92)
Recurrent pilonidal sinus	1 (1.96)
Flap necrosis	1 (1.96)
Epidermolysis	1 (1.96)

The recurrence of pilonidal sinus in one of our patients could be attributed to excessive body hair and underwent rhomboid flap surgery for the second time with good outcome. The case with flap necrosis refused the repeat rhomboid flap surgery and was managed with split skin graft. All other complications had good outcomes with conservative management.

The demographic variables are presented (Table 3).

**Table 3:** Demographic variables.

Variables	Value
Age (year)	38.73±19.47
Gender	
Male	30 (58.8%)
Female	21 (41.2%)

## DISCUSSION

The Limberg Rhomboid flap has been used successfully in repairing the cutaneous defects due to malignancy, bed sores, pilonidal sinus and traumatic ulcers as evidenced by past studies.^4–8^ In our study as well we have used rhomboid flap for similar cases. We have used rhomboid flap to cover the cutaneous defect by creating a similar shaped flap of equal size by mobilizing the lax skin of same texture and skin color.

In our study the complications were present in 8 (15.7%) cases which is higher than the study done by Alvarez et al. where they had only 8% complication rate.^5^ It can be explained on the basis of the patient selection, as 84% of the cases in their study were due to malignancy i.e. less numbers of bedsores and infected cases like pilonidal sinus.

Our complication rate in patients treated with rhomboid flap for bed sore is comparable with the study done by Bhatia et al. with the complications rate of 12%.^9^

In study done by Bozkurt et al. in pilonidal sinus management by rhomboid flap, the total complication rate was 12.5% which is comparable to our study.^10^

Likewise in study done by Arumugam et al.^11^ in pilonidal sinus the wound infection rate is 13% and recurrence rate is 7% which is higher than ours. The probable reason being the case selection; as their patients had multiple abscess drainage prior to surgery and the other reason is probably due to the location of the lower pole of the rhomboid at the midline of the natal cleft.^11^

In developing countries like Nepal, where the super specialty as plastic surgeon is not easily available, general surgeons are correcting such defects using limberg rhomboid flap. This technique is safe, easy to learn, versatile with minimal complications. Moreover the flap is easy to design, easy to use with good outcome. Since our study was conducted in a tertiary care hospital, generalizations of the results from the study cannot be done.

## CONCLUSIONS

The Limberg rhomboid flap can be used safely in patients with cutaneous defect with minimal complications and good surgical outcome however prevalence of complications after limberg rhomboid flap in patients with cutaneous defects at tertiary care center is high compared to the previous studies done.

## Conflict of Interest

**None.**
